# A permutation testing framework to compare groups of brain networks

**DOI:** 10.3389/fncom.2013.00171

**Published:** 2013-11-25

**Authors:** Sean L. Simpson, Robert G. Lyday, Satoru Hayasaka, Anthony P. Marsh, Paul J. Laurienti

**Affiliations:** ^1^Department of Biostatistical Sciences, Wake Forest School of MedicineWinston-Salem, NC, USA; ^2^Department of Radiology, Wake Forest School of MedicineWinston-Salem, NC, USA; ^3^Department of Health and Exercise Science, Wake Forest UniversityWinston-Salem, NC, USA

**Keywords:** graph theory, connectivity, fMRI, small-world, neuroimaging, Jaccard, Kolmogorov-Smirnov

## Abstract

Brain network analyses have moved to the forefront of neuroimaging research over the last decade. However, methods for statistically comparing groups of networks have lagged behind. These comparisons have great appeal for researchers interested in gaining further insight into complex brain function and how it changes across different mental states and disease conditions. Current comparison approaches generally either rely on a summary metric or on mass-univariate nodal or edge-based comparisons that ignore the inherent topological properties of the network, yielding little power and failing to make network level comparisons. Gleaning deeper insights into normal and abnormal changes in complex brain function demands methods that take advantage of the wealth of data present in an entire brain network. Here we propose a permutation testing framework that allows comparing groups of networks while incorporating topological features inherent in each individual network. We validate our approach using simulated data with known group differences. We then apply the method to functional brain networks derived from fMRI data.

## Introduction

As brain network analyses have become popular in recent years, neuroimaging researchers often face the need to statistically compare groups of brain networks (Simpson et al., 2013). A rudimentary and commonly used approach to conduct such comparisons is to summarize each network by a univariate metric and compare such summary measures by a simple test (e.g., a *t*-test). Although this approach may be appealing for its simplicity, such methods are blind to the complex local connectivity patterns within the network. In fact, to the best of our knowledge, the network-based statistic (NBS) (Zalesky et al., 2010) and the exponential random graph modeling (ERGM) framework (Simpson et al., 2011, 2012) are the only methods that attempt to account for topological differences across networks. The NBS and other mass-univariate analyses are designed to localize which nodes or edges differ between groups of networks. Although these approaches allow local comparisons of connectivity patterns or nodal property differences, they are predicated on edge by edge (or node by node) comparisons and only subsequently aggregate the results of these comparisons to identify clusters of nodal or edge-based differences. In other words, they are not designed to compare or account for topological features inherent in the entire network of each subject. Contrastingly, the ERGM framework allows capturing a network's topological features, but group comparisons pose difficulties (Simpson et al., 2011). Thus, analysis methods are needed that enable the comparison of groups of networks while incorporating topological features inherent in each individual network.

In order to develop such an analysis, we can exploit the fact that brain networks often exhibit consistent organizations across subjects. For example, a number of studies have reported that nodes with particular characteristics (e.g., high degree) tend to coincide at the same spatial locations across subjects (Hagmann et al., 2008; Van Den Heuvel et al., 2008; Hayasaka and Laurienti, 2010; Moussa et al., 2011). Although the set of such nodes may not be exactly the same across subjects, there are large areas of overlap (Hagmann et al., 2008; Hayasaka and Laurienti, 2010). Furthermore, our recent study on network modules, or communities of highly-interconnected nodes, indicated that some building blocks of resting-state functional brain networks exhibited remarkable consistency across subjects (Moussa et al., 2012). It has also been shown that such consistent organizations differ under different cognitive states (Deuker et al., 2009; Moussa et al., 2011; Rzucidlo et al., 2013) or in different groups of subjects (Rombouts et al., 2005; Stam et al., 2007; Liu et al., 2008; Bassett and Bullmore, 2009; Meunier et al., 2009a; Burdette et al., 2010; Yuan et al., 2010). Thus, an analysis method sensitive to such differences in spatial locations or patterns can assess group differences at the network level (as opposed to the edge or nodal level). In this work we propose a group comparison framework within which we develop two analysis methods. The first focuses on whether or not the spatial location of key nodes or modules is consistent between groups of networks beyond inter-subject variations observed within each group. In other words, this method compares the consistency of network organization between groups. Our second proposed method compares the degree distributions (distribution of the number of connections each node has) between groups. Assessment of the degree distribution provides insight into the overall topology of brain networks (Barabasi and Albert, 1999).

As mentioned above, our first proposed test takes into account the consistency of key node sets within and between groups. We do so by summarizing similarities in node sets across multiple networks with the Jaccard index, a metric that quantifies similarity in partitions of a set (Meunier et al., 2009b; Joyce et al., 2010). The Jaccard index is then incorporated into a permutation testing framework, enabling the formal assessment of group differences at the network level. Permutation tests have been used extensively in neuroimaging data analyses because they allow use of a test statistic with an unknown distribution as is the case with the Jaccard index in our context (Bullmore et al., 1999; Nichols and Holmes, 2002; Hayasaka and Nichols, 2003, 2004; Nichols and Hayasaka, 2003).

Our second proposed test compares the degree distributions within and between groups. We accomplish this by summarizing similarities in nodal cumulative degree distributions across multiple networks with the Kolmogorov-Smirnov statistic (K-S statistic), a measure that quantifies the distance between two cumulative distribution functions (Kolmogorov, 1933; Smirnov, 1948). As with the Jaccard Index, the K-S statistic is also incorporated into a permutation testing framework, enabling the assessment of statistical differences between groups of networks.

In this paper, we detail our *Permutation Network Framework* (PNF) for the Jaccard index (PNF-J) and K-S statistic (PNF-KS). We focus on the PNF-J, but introduce the PNF-KS as an additional useful approach. We validate the PNF-J using simulated data with known group differences in terms of locations of key nodes. We then apply both the PNF-J and PNF-KS to functional brain networks derived from fMRI data.

## Materials and methods

### Permutation test

A permutation test requires no knowledge of how the test statistic of interest is distributed under the null hypothesis (e.g., no significant group difference). This is because the permutation process empirically “generates” the distribution under the null hypothesis from the data by permuting data labels. Consequently, a permutation test can be a useful tool when the study design is simple (e.g., comparing two groups) yet the theoretical derivation of the test statistic is difficult. A group comparison of networks falls under such a scenario. Thus, we developed a framework to perform a permutation test for the comparison of groups of networks. It is important to note that permutation tests are not assumption free and do assume exchangeability (i.e., that when the null hypothesis is true the joint distribution of the observations remains unchanged under permutation of the group labels).

### Jaccard index

#### Identifying key nodes

**Figure 1:**
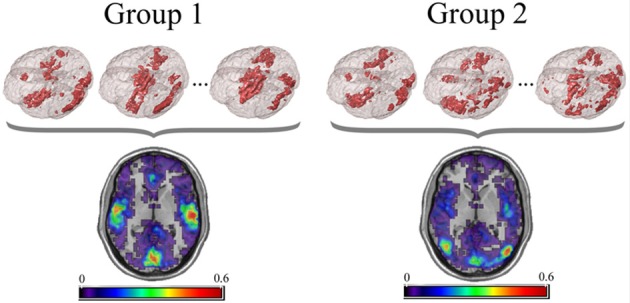
**Example visualization of key node sets from voxel-based networks in brain space**. The 3D brain images **(Top)** are 3 representative subjects from each group with the key node sets defined to be those with the top 20% highest degree. Overlap maps **(Bottom)** show the proportion of subjects with key nodes in given areas.

For the PNF-J, groups of network data having the same set of nodes can be compared. First, key nodes of interest in those networks need to be identified. Key nodes can be identified based on nodal characteristics such as high degree, high centrality, or other desired characteristics. Since those key nodes will be compared across subjects, it is important to employ the same criterion in all of the networks (e.g., top 10% highest centrality, node degree >200, etc.). Alternatively, key nodes can be identified as those belonging to a particular module, a collection of highly interconnected nodes. The resulting key nodes will form a set and the consistency of the spatial location of the nodes can be compared across groups. An example visualization of key node sets from voxel-based networks in brain space is given in Figure 1.

#### Establishing similarity/dissimilarity between networks

**Figure 2:**
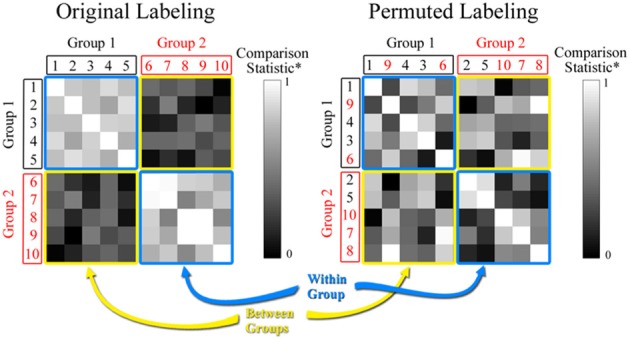
**Comparison statistic (Jaccard index and K-S statistic) matrix**. The figure shows hypothetical data for 2 groups each with 5 subjects. The value in each cell of the matrix represents the similarity between the two subjects based on either the Jaccard or the rescaled K-S statistics. The permuted matrix results in a scrambling of the group assignment for subjects based on random selection. Note that subjects 2 and 5 have been moved to Group 2 and subjects 9 and 6 have been moved to Group 1.

Key node sets likely include similar nodes within groups and different nodes between groups. To quantify how similar or dissimilar key node sets are between networks, we will employ the Jaccard index (Meunier et al., 2009b; Joyce et al., 2010). If *A* and *B* are the key node sets from two networks, then the Jaccard index between these networks can be calculated as

(1) $$J=\frac{\left|\text{A}\cap \text{B}\right|}{\left|\text{A}\cup \text{B}\right|},$$

where |A∩ B| is the number of key nodes that overlap in networks *A* and *B*, and |A∪ B| is the total number of key nodes in the two networks. *J* ranges from 0 (no overlap) to 1 (perfect overlap), with a large number indicating high similarity between the sets *A* and *B*. If there are multiple networks in a data set then *J* can be calculated for every pair in the data set, resulting in a matrix as can be seen in Figure 2. In the comparison statistic matrix in Figure 2, each row and column represents an individual network, and the (*i*, *j*)th element corresponds to the comparison statistic value (the Jaccard index in this case) between networks *i* and *j*. The main diagonal elements in the matrix are 1, since they represent *J* between identical key node sets. If there are two distinct groups among the networks, then the matrix includes two types of patterns. *J* is elevated among networks belonging to the same group since their key node sets are similar (note the blue squares in Figure 2). Conversely, *J* is attenuated if networks belong to different groups (note the yellow squares in Figure 2). Thus, the mean of *J* within groups is expected to be larger than the mean of *J* between groups. To summarize this disparity we define the Jaccard ratio *R*_J_ as

(2) $$R_{J}=\frac{M_{J}(\text{Within})}{M_{J}(\text{Between})},$$

where *M*_*J*_ corresponds to the mean of *J*. If *R*_*J*_ »1, then the Jaccard index is greater within groups than between groups, indicating that the groups differ significantly in the consistency of the key node sets. Conversely, if *R*_*J*_ ≈ 1, then there is no evidence that the groups differ significantly in the consistency of the key node sets. One of the strengths of the Jaccard ratio *R*_*J*_ is that it can account for inter-subject variations in key node set consistency within groups, while enabling an assessment of the between group differences in relation to such intra-group variations. The impetus for this *R*_*J*_ test statistic (and the *R*_*KS*_ statistic discussed in the next section) was a desire to create an ANOVA F-test analogue for our context. The main difference with our statistic is that averages across network relationships (key node set consistency and degree distribution similarity) are computed as opposed to averages across networks themselves.

### K-S statistic

Degree distributions, which help quantify the topology of networks, are likely more similar within distinctive groups than they are between these groups. We employ the K-S statistic noted in the Introduction to quantify this potential dissimilarity. If *F*_1_(x) and *F*_2_(x) are the cumulative degree distribution functions for two networks, then the K-S statistic for the difference between these distributions is

(3) $$KS=\underset{x}{\sup}|F_{1}(x)-F_{2}(x)|,$$

where *sup*_*x*_ is the supremum of the set of distances between distributions. *KS* ≥ 0, with larger values indicating more dissimilar distributions. As with the Jaccard index, *KS* can be calculated for every pair of networks in a multiple network data set to produce a matrix like the one shown in Figure 2. For visual purposes, we present rescaled *KS* values in Figure 2 [Rescaled *KS* = 1−(*KS*−KS_min_)/(*KS*_max_− KS_min_)] to align it with the range of the Jaccard index. Again each row and column represents an individual network, and the (*i*, *j*)th element corresponds to the rescaled *KS* value between networks *i* and *j*. The main diagonal elements in the matrix are 1, since they represent rescaled *KS* between the same distributions. If there are two distinct groups among the networks, then *KS* is relatively small (or relatively large for rescaled *KS*) among networks belonging to the same group (note the blue squares in Figure 2) and relatively large (or relatively small for rescaled *KS*) among networks belonging to different groups (note the yellow squares in Figure 2). Thus, the mean of *KS* within groups is expected to be smaller than the mean of *KS* between groups. To summarize this disparity we define the K-S ratio *R*_*KS*_ as

(4) $$R_{KS}=\frac{M_{KS}(\text{Between})}{M_{KS}(\text{Within})},$$

where *M*_*KS*_ corresponds to the mean of *KS*. If *R*_*KS*_ »1, then the K-S statistic is greater between groups than within groups, indicating that the groups differ significantly in their degree distributions. Conversely, if *R*_*KS*_ ≈ 1, then there is no evidence that the groups have significantly different degree distributions. Like the Jaccard ratio, the K-S ratio *R*_*KS*_ accounts for inter-subject variations (in degree distributions) within groups, while enabling an assessment of the between group differences in relation to such intra-group variations.

### Statistical assessment through permutations

The distributions of the Jaccard and K-S ratios (*R*_*J*_ and *R*_*KS*_) are unknown under the null hypothesis of no group difference. However, the null distribution can be generated empirically through a permutation process. In a two-sample comparison setting, this is done by randomly re-assigning, or permuting, group labels. This process, consisting of permuting group labels and re-calculating *R*_*J*_ or *R*_*KS*_, is repeated a large number of times (*L*_perm_). Table 1 describes the permutation process.

**Table 1:** **Steps involved in the permutation process**.

**Step**	**Procedure**
1	Calculate *R*_J/KS_ based on the original group labels
2	Randomly permute group labels
3	Re-calculate *R*_J/KS_ based on the permuted group labels from Step 2
4	Record *R*_J/KS_ from Step 3 as *R*_J/KS_^perm^
5	Repeat steps 2-4 *L*_perm_ times

**Figure 3:**
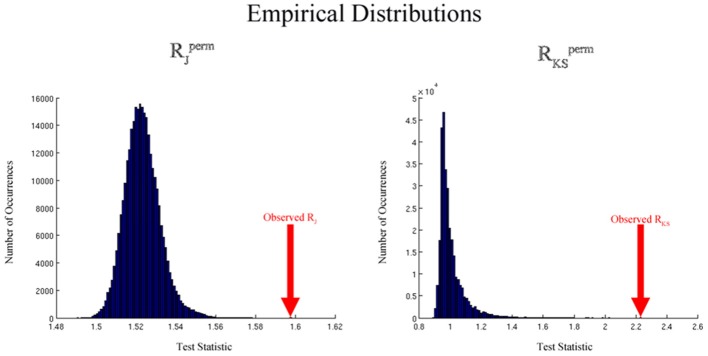
**Empirical distributions generated by the permutation process for the Jaccard (left) and K-S (right) analyses using simulated data**. The distributions are based on simulated data and *L*_perm_= 300,000 permutations. For the Jaccard data, simulated network degree images were used as described in the simulated data section. For the K-S data, simulated degree distributions were drawn from exponentially-truncated power law distributions.

This process results in a collection of *R*_*J*/*KS*_^perm^ values calculated under permuted group labels. We can determine the null distribution of *R*_*J*/*KS*_ by constructing the empirical distribution of *R*_*J*/*KS*_^perm^ values. Figure 3 shows distributions generated by the permutation methodology that are typical for the Jaccard and K-S analyses. The distributions are based on simulated data and 300,000 permutations. For the Jaccard data, simulated network degree images were used as described below in the Simulated Data section. The K-S permutation process was based on simulated degree distributions that were drawn from exponentially-truncated power law distributions (data not shown). By comparing *R*_*J*/*KS*_, the Jaccard or K-S ratio based on the original labels, to this empirical distribution, we can determine the statistical significance of the group difference. In particular, the *p*-value for this comparison can be computed as

(5) $$p=\frac{\#(R_{J/KS}{}^{\text{perm}}>R_{J/KS})}{N_{\text{perm}}},$$

where #(*R*_*J*/*KS*_^perm^>R_J/KS_) is the number of permutations for which *R*_*J*/*KS*_^perm^ is larger than *R*_*J*/*KS*_. If the *p*-value is small, it indicates that the observed ratio *R*_*J*/*KS*_ did not occur by chance alone, signifying strong evidence against the null hypothesis.

The permutation process described above is computationally efficient since it only involves re-ordering the rows and columns of the comparison statistic matrix based on the permuted group labels. Unlike permutation tests used in mass-univariate analyses of brain imaging data (Holmes et al., 1996; Bullmore et al., 1999; Nichols and Holmes, 2002), our permutation process does not involve the re-calculation of network statistics or imaging data. Thus, once the comparison statistic matrix is calculated, the permutation process can be accomplished with a relatively small computational burden, enabling a large number of permutations. The larger the number of permutations, the more precise the empirical *p*-value becomes. It should be noted, however, that the number of possible permutations is limited by the number of networks (study participants) in the data set. For example, if there are *N*_1_ and *N*_2_ networks in groups 1 and 2, respectively, then the number of possible permutations of group labels, *L*_max_, is given by

(6) $$L_{\max}=\left(\begin{array}{c} N_{1}+N_{2}\\ N_{1} \end{array}\right)=\frac{(N_{1}+N_{2})!}{N_{1}!N_{2}!}$$

For sufficiently large *N*_1_ and *N*_2_, it is not feasible to perform all possible permutations of group labels. Thus, in practice, only a subset of all possible permutations is used, with *L*_perm_ « *L*_max_. In this subset context, the computed *p-values* may slightly underestimate the “true” *p*-value by around 1/*L*_perm_, which is negligible in our case (around 3 × 10^−6^−1 × 10^−5^). However, this issue becomes problematic in mass-univariate settings in which multiple testing approaches can lead to inflated family-wise type I error rates. Exact tests have been developed to address this limitation (Phipson and Smyth, 2010).

### Paired two sample test

The permutation test framework described above is designed for comparing two independent groups of networks, but the framework can also be extended to paired two-sample tests. In a paired setting, two networks originate from the same subject. For example, two networks may represent brain networks before and after a certain treatment or intervention. Or, two networks may be constructed from neuroimaging data under two distinct cognitive conditions or states. Since each network in one group is “paired” with a network in the other group, the permutation procedure for two independent groups as described above cannot be directly applied in this setting. This is because the pairing information needs to be maintained during the permutation process. To do so, rather than randomly permuting group labels as is done in the independent two-sample test, the group label is flipped within each subject. For example, if each subject has two networks acquired under conditions X and Y, then these group labels may be permuted for this subject from XY to YX. Flipping the labels within each subject ensures that each subject's networks are represented in both groups. The flipping of the group label can be done for a random subset of the subjects in the data set. If there are *N* subjects in the data set, then there are 2^N^ possible permutations of group labels for the paired-test setting. Under the original labeling, as well as permuted labeling, the Jaccard and K-S ratios *R*_*J*/*KS*_ or *R*_*J*/*KS*_^perm^ can be calculated in the same way as described above. The statistical significance of a paired-test can also be calculated by comparing *R*_*J*/*KS*_ to the distribution of *R*_*J*/*KS*_^perm^ as described above.

## Simulation studies

### Data

**Figure 4:**
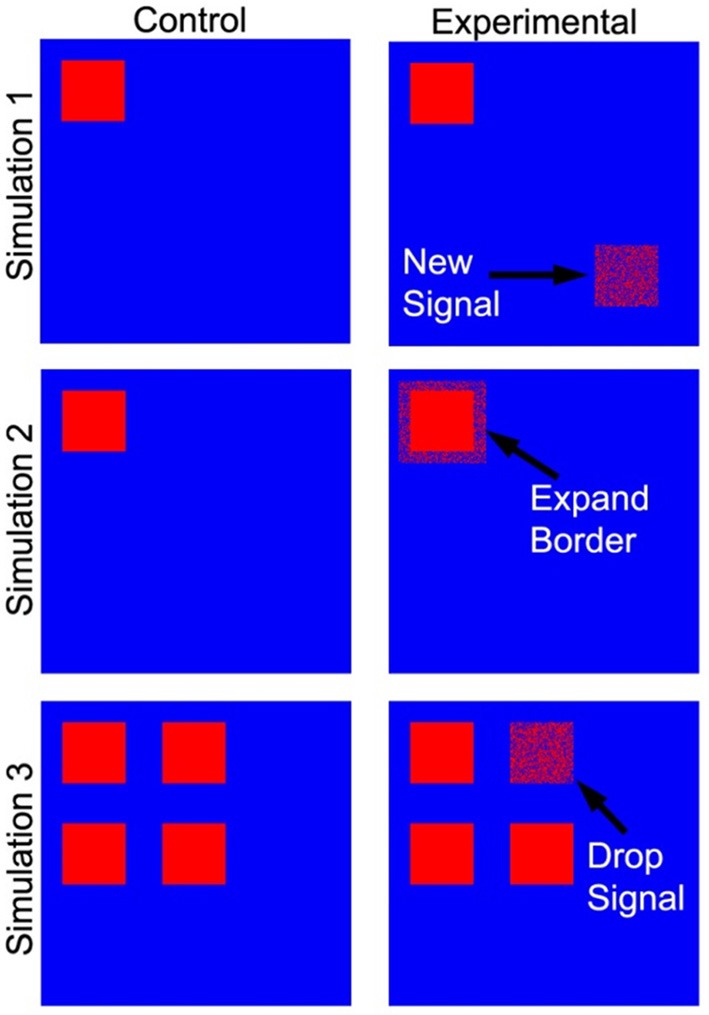
**Simulated data used as a ground truth**. The cartoon demonstrates the locations of the regions that were added (simulation 1), expanded (simulation 2), and dropped (simulation 3).

Three tests of significance based on simulated data (Figure 4) were performed to validate our PNF-J approach and show its ability to statistically identify various qualitative changes. For brain network analyses it is very common to qualitatively and quantitatively evaluate the locations of high degree network nodes. The first simulation was used to detect a new region of high degree nodes. The second simulation was used to detect an increase in the size of a region of high degree nodes. The third simulation was designed to identify the loss of a region of high degree nodes.

Each simulation contained two groups of ten subjects (control and experimental), and each subject's network contained 5400 nodes. For each node a value of one represented signal or noise (i.e., a true high degree node or falsely identified high degree node), while the absence of either was represented by zeros. The control groups for the first and second simulations contained a 216 node subset (region) with each node given a 40% probability of being defined as a signal. The remaining nodes were given a 10% probability of being defined as noise. For the experimental group in the first simulation, signal was introduced in a new 216 node subset of the network. Signal probability within this second subset was gradually increased across 10 iterations from 10 to 40%. As signal probability increased, it was expected that the control and experimental groups would become less similar. For the experimental group in the second simulation, the original 216 node subset (with 40% probability of signal) was increased to a 384 node subset. The signal probability in the additional nodes was gradually increased from 10 to 40% across 10 iterations. The goal of the third simulation was to assess test performance with a gradual reduction in signal. The control group contained four 216 node subsets of elements with each node in the subsets given an 80% probability of being defined as a signal. The experimental group was represented in the same way. However, the probability of signal in the nodes of one subset was gradually decreased from 80 to 50% while the signal in the other subsets remained constant.

### Results

**Figure 5:**
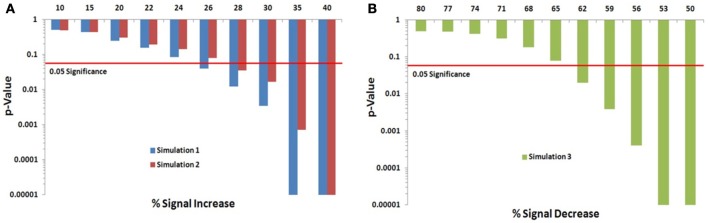
**Simulation results**. Note that when the *p*-value equals 0 it is set to the minimum value on the graph. Table A1 contains the actual values. **(A)** For the first simulation (blue), the significance threshold was first crossed when the second signal probability reached 26% (*p* = 0.0391). For the second simulation (red), the test first detected a significant signal expansion at a signal probability of 28% (*p* = 0.0354). **(B)** A signal probability reduction of 18% was detectable in the third simulation with the probability reduced to 62% from 80% (*p* = 0.0196).

We assessed the Permutation Network Framework for the Jaccard Index (PNF-J) with the three simulations detailed in the last section. Each simulation was run 1000 times and the average *p*-values across these runs were noted. Figure 5 (and Table A1) contains the results. The results of the first simulation demonstrate that the experimental group became more statistically different than the control group as the probability of the new signal was increased. The significance threshold (*p* = 0.05) was first crossed when the second signal probability reached 26% (*p* = 0.0391). The results of the second simulation show this same pattern with the test detecting a significant signal expansion at a signal probability of 28% (*p* = 0.0354). A signal probability reduction of 18% was detectable in the third simulation with the probability reduced to 62% from 80% (*p* = 0.0196).

**Figure 6:**
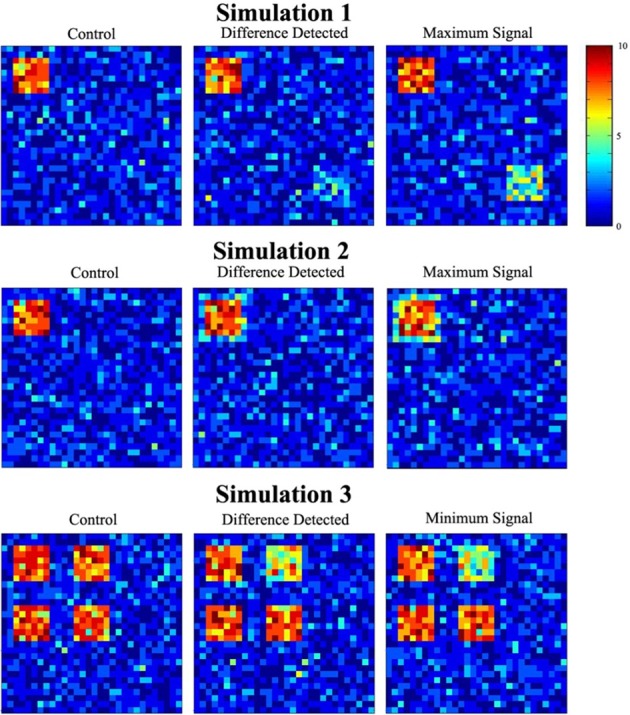
**Sample simulated data**. The figure shows a single slice through the overlap of the 10 simulated datasets. The overlap images show the number of individuals (color scale) that had signal at any particular location.

Figure 6 depicts data used in the three simulations. Each image is a cross-section of the overlap image created by summing the 10 binary images from the experimental and control groups (from one of the thousand trials). The value of each cell, ranging from 0 to 10, shows how many images had a signal at that location. Images in the first column show a cross-section of the control group overlap data for each simulation. The second column contains a slice of the experimental group overlap data for the minimum signal probability change at which significance was reached. Cross-sectional overlap images for the experimental group when the maximum signal probability change occurred are displayed in the last column.

## Experimental studies

### Data

The data used to apply and show the utility of the Permutation Network Framework (PNF) consist of fMRI scans from three separate datasets. The first dataset is part of the 1000 Functional Connectome Project available at (http://fcon_1000.projects.nitrc.org/). The dataset is from Leipzig consisting of subjects aged 23–42 years old (*n* = 37, male/female = 16/21). The data were collected during resting state with fixation on a cross. The Leipzig data was randomly divided into two groups (*n* = 18 and *n* = 19) and a two sample PNF-J test was performed.

The second dataset, the Aging Brain, was originally collected for a brain perfusion study (Hugenschmidt et al., 2009) and analyzed with both the PNF-J and PNF-KS. The Aging Brain dataset is populated by two groups, young subjects aged 27 ± 5.8 years old (*n* = 20), and old subjects aged 73 ± 6.6 years old (*n* = 19). Subjects were healthy and matched for gender and education. Three separate conditions of fMRI scans were used, resting, visual, and multisensory (MS) (visual and auditory), each lasting 5.6 min. The details of these conditions along with additional network analysis can be found in a previous publication (Moussa et al., 2011). For each fMRI scan, blood-oxygen-level dependence (BOLD) contrast was measured using a 1.5T MRI scanner and a whole-brain gradient echo-planar imaging (EPI) sequence with the following parameters: 200 volumes with 24 contiguous slices per volume; slice thickness = 5.0 mm; in-plane resolution of 3.75 mm × 3.75 mm; TR = 1700 ms.

The third dataset was from a group of eight older adults (male/female = 2/6, average age = 85.63) recruited for a physical/cognitive intervention. The data used here are all baseline data collected before the intervention. The data have not been published elsewhere. Three fMRI scans were performed in the same imaging session. The first was a resting scan, the second was a working memory scan (n-back), and the third was a repeat resting scan. Resting scans were performed while participants were viewing a fixation point on a screen. The working memory task was presented on a screen and participants responded by pressing a button with their right index finger if the letter being viewed matched the prior letter that was presented. They pressed the button with their middle finger if the letter did not match the prior letter. Letters were presented with an inter-stimulus interval of 3 s. All scans lasted 5:14 min and the first 14 s was discarded to allow the tissue magnetization to achieve steady-state. For each fMRI scan, BOLD contrast was measured on a 3T scanner using a whole-brain gradient echo- planar imaging (EPI) sequence with the following parameters: 157 volumes with 35 contiguous slices per volume; slice thickness = 5.0 mm; in-plane resolution of 3.75 mm × 3.75 mm; *TR* = 2000 ms.

### Data processing and network generation

All datasets utilized the same processing and network generation steps. To process the data, functional scans were normalized to standard brain space with a 4 × 4 × 5 mm voxel size. Data were band pass filtered (0.00765–0.068 Hz), and motion parameters, global signal, and mean WM and CSF signals were regressed from the imaging data.

In order to generate the networks, a correlation matrix was made for each subject containing the pairwise correlation coefficients between the time courses of all voxel pairs. Unweighted, undirected networks were then created by thresholding the correlation matrices for each subject to yield a set of adjacency matrices (**A**_ij_) with 1 indicating the presence and 0 indicating the absence of an edge between two nodes. Thresholding approaches generally fall into three categories (*fixed threshold*, *fixed degree*, *fixed edge density*) with there being no consensus on the best approach. We find the *fixed edge density* (or *wiring cost*) approach most appealing given that it avoids the confounding that can occur in subsequent analyses with networks that have widely varying degrees or connectivity distributions (Simpson et al., 2013). More specifically we used the method of Hayasaka and Laurienti (2010), which falls within this category, and defined (thresholded) all networks so that the relationship (denoted by S) between the number of nodes *n* and the average node degree *K* was the same across networks. In particular, the networks were defined so that S = log(n)/log(*K*) = 2.5 as this value has been show to lead to more stable and robust results (Hayasaka and Laurienti, 2010).

### Results

#### Two sample Jaccard test

**Figure 7:**
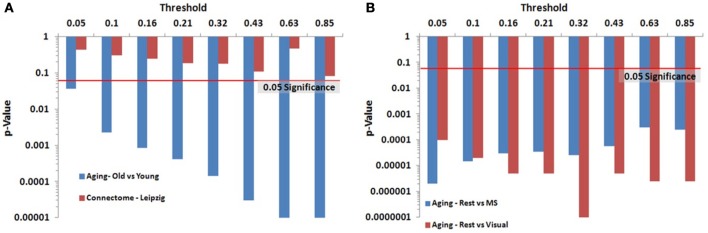
**Leipzig and Aging Brain data analysis results for the PNF-J**. Note that when the *p*-value equals 0 it is set to the minimum value on the graph. Table A2 contains the actual values. **(A)** The unpaired (independent) two sample tests show that there was a significant difference between younger and older adults in the Aging dataset but no difference in the two randomly selected groups from the Leipzig dataset. **(B)** The paired (dependent) two sample tests show that there were significant differences when comparing resting state with multisensory scans (MS) and resting state with visual scans of the same subjects in the younger group from the Aging dataset.

We identified key nodes of interest based on node degree, selecting the top 20% highest degree (hub) nodes (though the actual percentage varied some due to true nodal degree). However, a range of thresholds (selection percentages) was employed and those results are presented in Figure 7 (and Table A2) for the paired and unpaired two sample tests for the Leipzig and Aging Brain analyses. The results demonstrated that the method is robust to the specific chosen threshold. Note that when a threshold of 1 (100%) is applied to the data, the *p*-value will necessarily equal 1 since all nodes are selected as key nodes for each network.

For the first analysis, we created two randomly selected groups from the Connectome - Leipzig data. The two sample PNF-J test demonstrated that the two groups were not significantly different (*p* = 0.1864, threshold = 0.2138). This was expected given that each group was randomly drawn from the same sample population.

The second analysis, using the Aging Brain dataset, compared a group of older adults and younger adults during a resting state. Our method found the two groups to be significantly different (*p* = 5.00 × 10^−6^, threshold = 0.2144). This corroborates the age-related differences in brain topology found by many others (Gaal et al., 2009; Gong et al., 2009; Meunier et al., 2009a), but our approach provides even more confidence in these differences given the size of the *p*-value. We also conducted two paired two-sample tests on the young group in the same dataset comparing resting state with multisensory scans (MS) and resting state with visual scans of the same subjects. These tests showed that when visual (*p* = 3.5 × 10^−5^, threshold = 0.2131) and multisensory (*p* = 1.0 × 10^−5^, threshold = 0.2138) information is being processed, significantly different topological configurations result.

**Figure 8:**
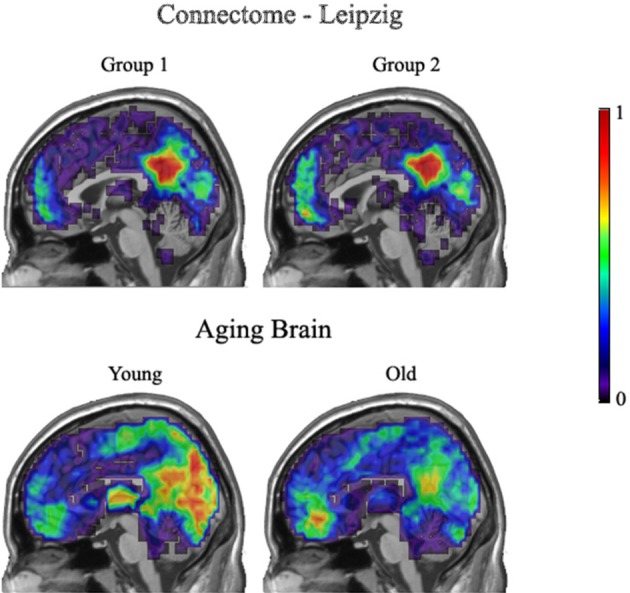
**Leipzig and Aging Brain analysis results**. Each image is a sagittal slice of an overlap of every member in a group at a threshold of 20%. The images are normalized by dividing by the number of subjects in each group, resulting in values between zero and one.

Figure 8 demonstrates group overlap maps for the Leipzig data and the Aging Brain young and older adults' data groups. Each image is a sagittal slice of an overlap of every member in a group at a threshold of 20%. The images are normalized by dividing by the number of subjects in each group, resulting in values between zero and one. Thus, the color scale shows the percentage of subjects that had a key hub node in any particular brain location. The first row contains resting state images from the Leipzig subset of the Connectome project. As stated above, the two groups proved to not be significantly different.

The second row shows the young and older groups of the Aging Brain dataset. The group differences that achieved significance are visually apparent. The main points of distinction between the young and older groups are in the precuneus and visual cortices where the young group has a stronger concentration of voxels above the 20% threshold across all subjects. In addition, the older adults had a higher concentration of hubs located in the medial inferior frontal lobe.

**Figure 9:**
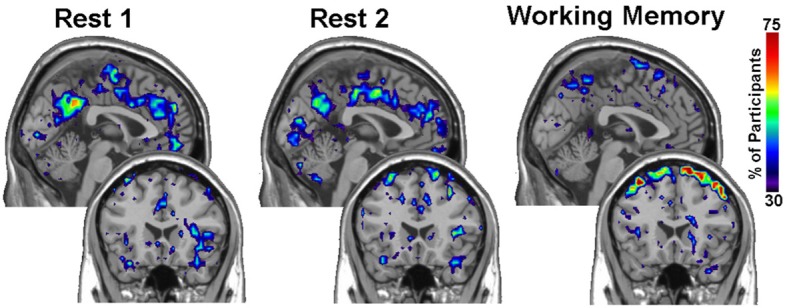
**Third study analysis results**. The images are sagittal **(top row)** and coronal **(bottom row)** slices of an overlap of every member in each condition at a degree threshold of 20%. The images show the percent of participants (color scale) that had hub nodes at any particular location.

**Figure 10:**
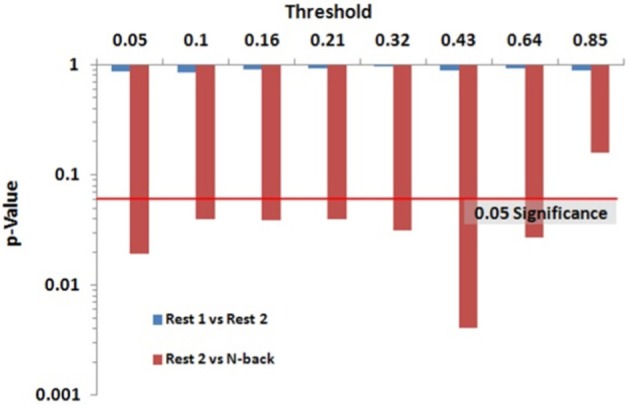
**Third study data analysis results for the PNF-J**. The test revealed a significant difference between Rest 2 and *N*-back (red) at the specified threshold (*p* = 0.0396, threshold = 0.21) but no difference between Rest 1 and Rest 2 (blue) (*p* = 0.93, threshold = 0.21).

Within group differences were evaluated in the data from the third study, again by comparing the spatial consistency of the top 20% highest degree (hub) nodes. The first analysis compared the overlap of hub nodes between the first and second resting state scans. The images showing the location and consistency of these hubs in the two conditions are shown in Figure 9. The images qualitatively demonstrate the consistency of hub locations across the two groups. The PNF-J test corroborated this qualitative finding, failing to identify a significant difference between the two resting conditions (*p* = 0.93, threshold = 0.2146). In fact, the comparisons between the two resting conditions did not achieve significance for any threshold tested. The second analysis compared the spatial consistency of network hubs at rest to those observed during the working memory task. The two conditions resulted in quite distinct hub patterns as shown in Figure 9. The PNF-J test comparing these maps revealed a significant condition difference (*p* = 0.0396, threshold = 0.2133). The significant difference held for all thresholds less than 84%. The results for all thresholds tested for both comparisons are shown in Figure 10 (and Table A3). These paired sample test results reveal that differences exist in the spatial location of high degree nodes between conditions but not when comparing two identical conditions.

#### Two sample K-S test

**Figure 11:**
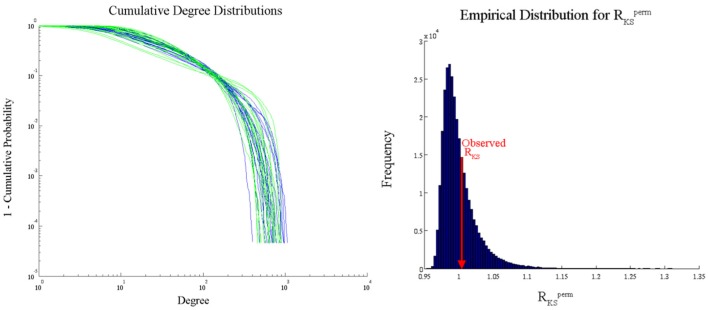
**PNF-KS results for Aging Data—young vs. older adults during a visual task (ACC only)**. Distribution plots **(left)** show the cumulative degree distributions for the young (blue) and older (green) adults. The empirical distribution of *R*_*KS*_^perm^ **(right)** was generated based on *L*_perm_ = 100.000 permutations. 35.58% (*p* = 0.3558) of the *R*_*KS*_^perm^ values were greater than the observed *R*_*KS*_ value.

We analyzed the Aging Brain dataset degree distributions with the PNF-KS approach in order to compare the results with those from conducted comparisons of parameters based on fits to an exponentially truncated power law. Degree distributions were fitted with an exponentially truncated power law using the following equation [*P*(*k*) = k^−β^ exp(−k/θ)] via maximum likelihood estimation based on prior work from our research group (Hayasaka and Laurienti, 2010). The parameter estimates, θ_est_ and β_est_, were then used to statistically compare the distributions between groups and across conditions via two-sample *t*-tests. A significant difference was found between the degree distributions of the young and older adults only during the visual state in the anterior cingulate cortex (ACC) (*p* = 0.01). However, this parametric analysis makes a strong distributional assumption, namely that the true degree distributions in young and older adults follow an exponentially truncated power law, which can lead to erroneous conclusions if the assumption is not met. We used our PNF-KS method, which makes no distributional assumptions, to substantiate this finding and found no evidence for a distributional difference in the ACC (*p* = 0.3558). Figure 11 displays the actual cumulative degree distributions and the results of this comparison between the young and older adults (ACC only) during the visual state (empirical distribution of *R*_*KS*_^perm^ and observed *R*_*KS*_). Given the overlap of the distributions in the two groups, it appears that our PNF-KS approach likely results in the appropriate conclusion while the parametric approach likely leads to a false positive finding.

## Discussion

The explosion of brain network analyses over the last decade has led to many important findings, but has also created many methodological gaps. Here we filled one of those gaps by providing a framework that allows accounting for spatial and topological information when comparing groups of whole-brain networks. Moreover, our Permutation Network Framework (PNF) makes no assumptions about the distribution of the test statistics of interest which often have non-standard distributions due to the complex nature of the data. We detailed two analysis methods embedded within this framework, namely the PNF-J and PNF-KS. Our focus was on the PNF-J, with the PNF-KS approach added to specifically analyze the Aging Brain dataset and compare the results with those obtained based on a parametric analysis. Addition of the PNF-KS also highlighted the fact that our framework allows the implementation of other modified statistics. Simulation studies (for the PNF-J) and analyses of fMRI data (for the PNF-J and PNF-KS) exhibited the utility and appeal of our approach.

Unlike current network comparison methods, the PNF-J and PNF-KS allow assessing differences between groups at the network level, as opposed to the edge or nodal level. The PNF-J enables comparing the spatial consistency of topologically important areas of the brain (key node sets) across groups. Its greatest appeal lies in the fact that the key node set selection can be informed by a specific question of interest, allowing an alignment of the research goals with the analytical methods employed. For example, a researcher might be interested in whether a stroke in a particular location would have a differential impact on different groups. Groups with hub nodes that are consistently concentrated in that location will suffer more severe damage than those with spatially inconsistent or more widely distributed hubs. Employing the PNF-J, with hubs as the key nodes, allows distinguishing these groups. The PNF-KS enables discerning whether degree distributions differ between groups, thus giving further insight into the capacity of localized brain injury or degeneration to affect overall brain capabilities in distinct populations. Most importantly, our Permutation Network Framework is easily implementable, as outlined in the Methods section, and provides a tool to extract meaningful information from complex data.

Network analyses provide complementary insight to the traditional activation localization analyses. The network based paradigm takes the perspective that many diseases don't just affect specific brain regions but that they affect patterns of connectivity across the brain. Network based approaches like ours aim to detect these topological differences and provide insight into the spatial distribution of these differences via the presented overlap maps. While the methods described here are powerful and yield test statistics without reducing the networks to summary metrics, there are some limitations to the techniques. In particular, the use of the PNF-J to compare hub nodes (or other key node sets) simply determines if (and where) the spatial locations of the hubs are different between groups. It does not indicate if the number of connections or the regions that the hubs are connected to are different between groups. In order to make such determinations, additional *post hoc* analyses are required. Once a group difference is identified using the permutation testing framework proposed, the *post hoc* analyses are then better justified and can be used to explore and explain network properties that underlie this observed statistical difference. Another potential limitation of our approach, mentioned in the “Statistical Assessment Through Permutations” subsection, is that inflated family-wise type I error rates may result if used in a massively multiple testing context. In this case, exact tests like those of Phipson and Smyth (2010) should be incorporated.

Brain network analysis is ripe for methodological development and our method provides a useful addition to the suite of whole-brain network comparison tools. Brain network analyses hold great potential for aiding in our understanding of normal and abnormal brain function. However, to realize this potential, we must continue to conscientiously develop tools that account for and exploit the complexity in the data.

## Conflict of interest statement

The authors declare that the research was conducted in the absence of any commercial or financial relationships that could be construed as a potential conflict of interest.

## Appendix

**Table A1:** **Simulation results**.

**Simulation**	**Signal probability (%)**	***P*-value**
1	10	0.5004
	15	0.4258
	20	0.2413
	22	0.1529
	24	0.0851
	26	0.0391
	28	0.0122
	30	0.0034
	35	0
	40	0
2	10	0.4914
	15	0.4355
	20	0.2989
	22	0.1929
	24	0.1392
	26	0.078
	28	0.0354
	30	0.0166
	35	0.0007
	40	0
3	80	0.4893
	77	0.479
	74	0.4094
	71	0.31
	68	0.1816
	65	0.0777
	62	0.0196
	59	0.0039
	56	0.0004
	53	0
	50	0

**Table A2:** **Leipzig and Aging Brain data analysis results for the PNF-J**.

**Test type**	**Dataset**	**Threshold**	***P*-value**
Unpaired two sample	Aging—old vs. young	0.0537	0.0363
		0.1074	0.0023
		0.1609	8.45 × 10^−4^
		0.2144	4.15 × 10^−4^
		0.3208	1.40 × 10^−4^
		0.4264	3.00 × 10^−5^
		0.6349	0
		0.8476	0
		1	1
	Connectome Leipzig	0.0535	0.4387
		0.1069	0.3073
		0.1604	0.2436
		0.2138	0.1864
		0.3206	0.1765
		0.4277	0.1066
		0.6395	0.4672
		0.8505	0.0828
		1	1
Paired two sample	Aging—rest vs. MS	0.0533	2.00 × 10^−5^
		0.1067	1.50 × 10^−5^
		0.1599	3.00 × 10^−5^
		0.2131	3.50 × 10^−5^
		0.3193	2.50 × 10^−5^
		0.4243	5.50 × 10^−5^
		0.6321	3.10 × 10^−4^
		0.8411	2.50 × 10^−4^
		1	1
	Aging—rest vs. visual	0.0535	9.80 × 10^−5^
		0.107	2.00 × 10^−5^
		0.1604	5.00 × 10^−6^
		0.2138	5.00 × 10^−6^
		0.3202	0
		0.4251	5.00 × 10^−6^
		0.633	2.50 × 10^−6^
		0.8415	2.00 × 10^−6^
		1	1

**Table A3:** **Third study data analysis results for the PNF-J**.

**Comparison**	**Threshold**	***P*-value**
Rest 1 vs. rest 2	0.0536	0.8677
	0.1074	0.8523
	0.161	0.9141
	0.2146	0.9347
	0.3211	0.9684
	0.4275	0.8904
	0.6404	0.9178
	0.8465	0.8866
	1	1
Rest vs. N-back	0.0534	0.0192
	0.1066	0.0394
	0.1597	0.0388
	0.2133	0.0396
	0.3196	0.0312
	0.4249	0.0041
	0.6332	0.0273
	0.8417	0.1587
	1	1
